# Microtubule *S*-glutathionylation as a potential approach for antimitotic agents

**DOI:** 10.1186/1471-2407-12-245

**Published:** 2012-06-15

**Authors:** Wei Chen, Teresa Seefeldt, Alan Young, Xiaoying Zhang, Yong Zhao, John Ruffolo, Radhey S Kaushik, Xiangming Guan

**Affiliations:** 1 Zhejiang Cancer Research Institute, Zhejiang Cancer Hospital, Hangzhou, Zhejiang, 310022, China; 2 Department of Pharmaceutical Sciences, South Dakota State University, Brookings, SD 57007, USA; 3 Department of Veterinary and Biomedical Sciences, South Dakota State University, Brookings, SD, 57007, USA; 4 ACEA Bio CO., Ltd., Hangzhou, Zhejiang, 310030, China; 5 Department of Physiology, Michigan State University, East Lansing, MI, 48824, USA; 6 Department of Biology and Microbiology, South Dakota State University, Brookings, SD, 57007, USA

## Abstract

**Background:**

Microtubules have been one of the most effective targets for the development of anticancer agents. Cancer cells treated by these agents are characterized by cell arrest at G_2_/M phase. Microtubule-targeting drugs are, therefore, referred to as antimitotic agents. However, the clinical application of the current antimitotic drugs is hampered by emerging drug resistance which is the major cause of cancer treatment failure. The clinical success of antimitotic drugs and emerging drug resistance has prompted a search for new antimitotic agents, especially those with novel mechanisms of action. The aim of this study was to determine whether microtubules can be *S-*glutathionylated in cancer cells and whether the glutathionylation will lead to microtubule dysfunction and cell growth inhibition. The study will determine whether microtubule *S-*glutathionylation can be a novel approach for antimitotic agents.

**Methods:**

2-Acetylamino-3-[4-(2-acetylamino-2-carboxyethylsulfanylcarbonylamino)phenyl carbamoylsulfanyl]propionic acid (2-AAPA) was used as a tool to induce microtubule *S*-glutathionylation. UACC-62 cells, a human melanoma cell line, were used as a cancer cell model. A pull-down assay with glutathione *S-*transferase (GST)-agarose beads followed by Western blot analysis was employed to confirm microtubule *S*-glutathionylation. Immunofluorescence microscopy using a mouse monoclonal anti-α-tubulin-FITC was used to study the effect of the *S-*glutathionylation on microtubule function; mainly polymerization and depolymerization. Flow cytometry was employed to examine the effect of the *S-*glutathionylation on cell cycle distribution and apoptosis. Cell morphological change was followed through the use of a Zeiss AXIO Observer A1 microscope. Cancer cell growth inhibition by 2-AAPA was investigated with ten human cancer cell lines.

**Results:**

Our investigation demonstrated that cell morphology was changed and microtubules were *S*-glutathionylated in the presence of 2-AAPA in UACC-62 cells. Accordingly, microtubules were found depolymerized and cells were arrested at G_2_/M phase. The affected cells were found to undergo apoptosis. Cancer growth inhibition experiments demonstrated that the concentrations of 2-AAPA required to produce the effects on microtubules were compatible to the concentrations producing cancer cell growth inhibition.

**Conclusions:**

The data from this investigation confirms that microtubule *S*-glutathionylation leads to microtubule dysfunction and cell growth inhibition and can be a novel approach for developing antimitotic agents.

## Background

Microtubules are involved in a diverse range of cellular functions, including motility, maintenance of cell shape, adhesion, intracellular trafficking of macromolecules and organelles, and, most importantly, mitosis [1-4]. The role that microtubules play in mitosis makes them one of the most highly validated targets for the development of chemotherapeutic drugs against rapidly dividing cancer cells [5]. Evidence has shown that subtle alteration in the structure of microtubules can cause improper attachment of chromosomes and impair the tension, which in turn signals the spindle checkpoint to prevent anaphase onset and chromosome segregation. The cell eventually exits mitosis aberrantly and undergoes apoptosis [5]. The effectiveness of microtubule-targeting drugs has been demonstrated by the extensive clinical use of several vinca alkaloids (vinblastine, vincristine, etc.) and taxanes (paclitaxel and docetaxel) for the treatment of a wide variety of human cancers [5]. Cancer cells treated with these agents are characterized by cell cycle arrest at G_2_/M phase. Microtubule-targeting drugs are, therefore, frequently referred to as antimitotic agents.

Structurally, microtubules are composed of similar 50 kDa α/β-tubulin heterodimers that share 40% sequence identity with almost identical three-dimensional structures [6]. Formation of microtubules involves reversible, noncovalent polymerization of repeating α/β-tubulin subunits that bind head to tail into protofilaments [7-9]. Tubulins are rich in thiol groups with a total of 20 free thiol groups per tubulin dimer (12 in α-tubulin and 8 in β-tubulin) [10,11]. These thiol groups are critical to tubulin polymerization or microtubule formation and function [12]. Loss of these thiol groups inevitably affects tubulin polymerization as observed in the oxidation [13,14] or alkylation [11] of these thiol groups. Mechanistically, most current antimitotic agents are classified into one of three classes based on their interactions with microtubules [15]. The first class of antimitotic agents, exemplified by paclitaxel, interact with the taxane binding site (M-loop) and cause microtubule stabilization [15]. The second class includes the vinca alkaloids which inhibit tubulin assembly by interacting at the interface between α,β-dimers resulting in microtubule depolymerization. The third class of compounds interact at the colchicine-binding site, the interface of the α,β-dimers, and prevent assembly of tubulin into microtubules [15].

**Figure 1:**
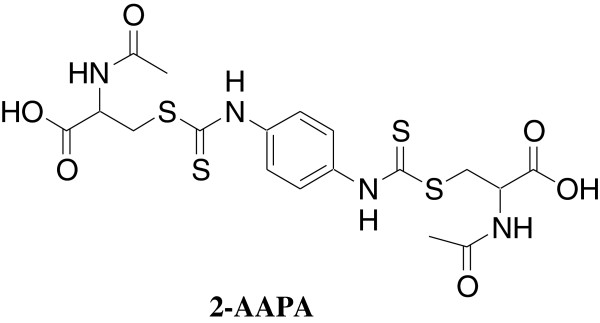
Chemical structure of 2-AAPA.

**Scheme 1:**
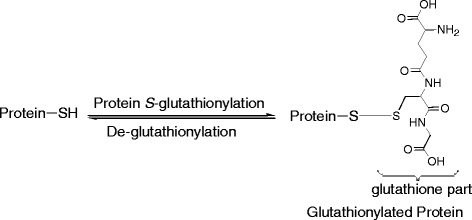
Protein S-glutathionylation.

In our earlier study with 2-acetylamino-3-4-(2-acetylamino-2-carboxyethylsulfanylcarbonylamino)phenylcarbamoylsulfanyl]propionic acid (2-AAPA, Figure 1) to modulate intracellular thiol oxidative stress, we found that 2-AAPA caused cell morphological change and cell detachment indicating that the cellular cytoskeletal structure was affected [16,17]. Further, 2-AAPA was shown to induce protein *S*-glutathionylation [16,17]. Protein *S*-glutathionylation is a process where a glutathione molecule (GSH) is connected to a protein thiol (PSH) through a disulfide bond (-S-S-) (Scheme 1) [13,18,19]. In view of the critical role thiol groups play in microtubule function and the rich content of thiol groups present in the microtubule structure, we suspected that 2-AAPA might induce microtubule *S*-glutathionylation and the *S*-glutathionylation might cause dysfunction of microtubules. If confirmed, microtubule *S-*glutathionylation could be a novel approach for the development of antimitotic agents which exhibit a different mechanism of action than the currently employed antimitotic drugs. These agents will be helpful in overcoming the emerging drug resistance problem associated with vinca alkaloids and taxanes. The aim of this study was to investigate whether microtubule can be *S*-glutathionylated in cancer cells and whether the *S*-glutathionylation leads to microtubule dysfunction and cell growth inhibition. 

## Methods

### Materials and cell lines

2-AAPA was synthesized as described previously [20]. 2-AAPA is currently also available from Sigma-Aldrich Chemical Co. (Milwaukee, WI). *p*-[3-(4,5-Dimethylthiazol-2-yl)-2,5-diphenyltetrazolium bromide] (MTT), ethylenediaminetetraacetic acid (EDTA), *N*-2-hydroxyethylpiperazine-*N*’-2-ethanesulfonic acid (HEPES), 2-amino-2-(hydroxymethyl)propane-1,3-diol hydrochloride (Tris–HCl) buffer, 4′,6-diamidino-2-phenylindole (DAPI), paclitaxel, vinblastine, propidium iodide (PI), RNase, horseradish peroxidase, sodium dodecyl sulfate (SDS), Triton-X-100, and mouse monoclonal anti-α-tubulin-FITC were obtained from Sigma-Aldrich Chemical Co. (Milwaukee, WI). The Vybrant™ Apoptosis Assay Kit #2 was purchased from Molecular Probes (Carlsbad, CA). Dulbecco’s phosphate buffered saline (DPBS), fetal bovine serum (FBS), minimum essential medium (MEM), phosphate buffered saline (PBS), RPMI-1640, and trypsin-EDTA were from Mediatech, Inc. (Herndon, VA). Dulbecco’s modified eagle’s medium (DMEM) was purchased from the American Type Culture Collection (ATCC). Sulfosalicylic acid was purchased from J.T. Baker Chemical Co. (Phillipsburg, NJ). Antibodies of α-tubulin and β-tubulin, secondary antibodies and GST-agarose beads were from Santa Cruz Biotechnology (Santa Cruz, CA). ECL Plus Kit was purchased from Amersham Biosciences (Piscataway, NJ). Other reagents were obtained in their highest purity grade available commercially. Cells were cultured in MEM, DMEM or RPMI 1640 growth medium supplemented with 10% FBS, 100 units/mL of penicillin (Mediatech, Inc., Herndon, VA) and 100 μg/mL of streptomycin (Mediatech, Inc., Herndon, VA) in a humidified atmosphere containing 5% CO_2_ at 37°C. Human cancer cell lines NCI/ADR-RES (ovarian cancer), OVCAR-3 (ovarian cancer), UACC-62 (melanoma), NCI-H226 (lung cancer), PC-3 (prostate cancer), and UO-31 (renal carcinoma) were obtained from the National Cancer Institute (NCI); A-431 (epidermoid carcinoma), SK-MEL-2 (melanoma), and MCF7 (breast cancer) were obtained from ATCC. GC_3_/c1 human colon adenocarcinoma cells were kindly provided by Dr. Peter J. Houghton of St. Jude Children’s Research Hospital, Memphis, Tennessee.

### *In vitro* cytotoxicity evaluation

2-AAPA was tested *in vitro* for cytotoxicity against NCI/ADR-RES, OVCAR-3, UACC-62, UO-31, NCI-H226, A431, MCF7, PC-3, SK-MEL-2 and GC_3_/c1 cells in 96-well plates using MTT assay to determine IC_50_ values. In brief, exponentially growing cells were plated in 96-well plates (1500, 2000, 1500, 2000, 1800, 1000, 2500, 2000, 2000 and 5000 cells/well for NCI/ADR-RES, OVCAR-3, UACC-62, UO-31, NCI-H226, A431, MCF7, PC-3, SK-MEL-2 and GC_3_/c1 cells respectively) in various media (RPMI 1640 for NCI/ADR-RES, OVCAR-3, UACC-62, UO-31, NCI-H226, PC-3, SK-MEL-2 and GC_3_/c1, MEM supplemented with 10 μg/mL insulin for MCF7, and DMEM for A431 respectively). The cells were allowed to attach at 37°C in a humidified atmosphere of 5% CO_2_ for 24 h, and then the medium was replaced with the medium containing various concentrations of 2-AAPA. Cells were exposed to the medium containing 2-AAPA for 6 days at the same incubation condition. After the treatment, MTT assay was performed to determine IC_50_ values.

### Determination of protein *S*-glutathionylation by HPLC

Protein samples from UACC-62 cells treated with 2-AAPA were prepared and *S*-glutathionylation was determined by the HPLC method described by Chen et al. [16].

### Determination of tubulin *S*-glutathionylation by a pull-down assay

Protein was extracted from UACC-62 cells using the method described by Chen et al. [16] with minor modification. Briefly, UACC-62 cells (5 million) were detached by trypsinization and treated with 100 μM 2-AAPA at 37^o^C for 20 min. After washing with ice-cold PBS, one milliliter of ice-cold sulfosalicylic acid aqueous solution (3%, w/w) was added to the cells. The lysed cells were centrifuged at 15000 × *g* for 1 min. The supernatant was discarded and the lysate pellets were washed thoroughly with the sulfosalicylic acid aqueous solution (1 mL × 3) to ensure that no residual non-protein thiols were left. Then the pellets were washed twice with Tris–HCl buffer (50 mM, pH 7.4) containing 5 mM EDTA. The pellets were collected by centrifugation at 15000 × *g* for 1 min and solubilized in 200 μL of denaturing lysis buffer with 1% SDS without dithiothreitol prepared as described by Bonifacino et al. [21]. The protein solution was then heated at 100°C for 5 min. The supernatant was collected after centrifugation at 15000 × *g* for 5 min and stored at −20°C for future use. For tubulin *S-*glutathionylation determination, 500 μg of protein sample was incubated with 40 μL of GST- agarose beads in a total of 1 mL of HEPES buffer containing 1% Triton X-100 overnight at 4°C on a platform rocker. GST-agarose beads were collected by centrifugation at 5,000 × *g* for 5 min at 4°C and gently washed with PBS followed by centrifugation to collect the beads. The wash was repeated four times. After the final wash, supernatant was carefully aspirated and discarded, and pellets were resuspended in 60 μL of 2× non-reducing electrophoresis sample buffer. Samples were boiled for 5 min at 100°C and centrifuged at 15000 × *g* at 4°C for 5 min. The *S*-glutathionylated tubulin proteins in the supernatant were subjected to SDS- PAGE. Then the proteins in gels were transferred to nitrocellulose membranes and probed with the appropriate dilution of primary α- or β-tubulin antibodies followed by appropriate horseradish peroxidase (HRP) conjugated secondary antibody and ECL Plus detection kit. The bands were visualized using a UVP Biochem Gel Documentation system (UVP, Inc., Upland, CA).

### Cell morphological change

Cultured UACC-62 cells were treated with 50 μM 2-AAPA at 37^o^C. Live cell images were taken by a Zeiss AXIO Observer A1 microscope equipped with a temperature-controlled stage at 37°C.

### Indirect immunofluorescence microscopy

UACC-62 cells were incubated with 2-AAPA at 37°C in a humidified atmosphere of 5% CO_2_ for various time periods and then fixed in 4% paraformaldehyde at room temperature for 1 h. The cells were washed thrice with PBS and incubated with cell permeable solution (0.1% Na-citrate, 0.1% Triton-X-100 in 1 × PBS) at room temperature for 1 h. After incubation with the blocking solution (PBS containing 5% bovine serum albumin) overnight at 4°C, microtubules were visualized using a mouse monoclonal anti-α-tubulin-FITC (1:1,000), and nuclei were stained with DAPI (1 μg/mL). Fluorescent images were taken with an Olympus AX70 fluorescence microscope. Paclitaxel and vinblastine were employed as positive controls for microtubule stabilization and microtubule depolymerization, respectively.

### Analysis of cellular DNA content by flow cytometry

UACC-62 cells (1 million) were plated in 100 mm culture dishes in RPMI 1640 medium for 24 h attachment at 37^o^C in a humidified atmosphere of 5% CO_2_, and then treated with 15 mL of RPMI 1640 medium containing various concentrations of 2-AAPA for 12 h or 24 h at the same incubation condition. At the end of treatment, adherent cells were harvested and washed twice with ice-cold PBS. One million cells of each sample were fixed with 70% ethanol in DPBS at 4°C. The fixed cells were then centrifuged (700 × g, 5 min) and washed with staining buffer. After the wash, the samples were centrifuged (700 × g, 5 min), and the pellets were treated with 100 μL of RNase A (1 mg/mL) for 30 min at 37°C. After incubation, 900 μL of staining buffer was added to the samples to bring the volume to 1 mL followed by addition of 20 μL of propidium iodide (PI) (1 mg/mL). The sample was then incubated in the dark at room temperature for 30 min before being analyzed with a BD FACScan™ flow cytometer (BD Biosciences, San Jose, CA) using CellQuest Software (BD Biosciences, San Jose, CA).

### Apoptosis assay by flow cytometry

UACC-62 cells (0.2 million) were plated in 25 cm^2^ flasks with RPMI 1640 growth medium. After a 24 h-attachment at 37^o^C in a humidified atmosphere of 5% CO_2_, UACC-62 cells were treated with RPMI 1640 growth medium containing various concentrations of 2-AAPA. Annexin V/PI staining was performed using the Vybrant™ Apoptosis Assay Kit #2 according to the manufacture’s instruction. The samples were analyzed with a BD FACScan flow cytometer.

### Statistical analysis

Data were analyzed with INSTAT software (Graph Pad, San Diego, CA). ANOVA followed by Tukey’s post test was applied to compare the statistical difference between 2-AAPA treatment groups and untreated controls. Significance in all experiments was considered at *P* < 0.05. Values were expressed as mean ± the standard deviation of the mean.

## Results

### *In vitro* cytotoxicity of 2-AAPA against various human cancer cells

The cell growth inhibition of 2-AAPA was evaluated with various human cancer cell lines. As demonstrated in Table 1, 2-AAPA exhibited similar IC_50_ values, in the range of 22 to 75 μM, against all the tested cancer cells revealing that the cytotoxicity is not cancer cell type-dependent. Based on the IC_50_ values, UACC-62 appears to be the most sensitive cells towards 2-AAPA and, therefore, was selected for further study.

**Table 1:** Anticancer activity evaluation of 2-AAPA against various human cancer cells

**Cell line**	IC50^*^(μM)
NCI/ADR-RES (ovarian cancer)	28 ± 1
OVCAR-3 (ovarian cancer)	33 ± 2
UACC-62 (melanoma)	22 ± 1
SK-MEL-2 (melanoma)	40 ± 9
NCI-H226 (lung cancer)	68 ± 4
UO-31 (kidney cancer)	44 ± 10
A431 (skin cancer)	73 ± 4
PC-3 (prostate cancer)	50 ± 12
MCF7 (breast cancer)	75 ± 5
GC_3_/c1 (colon cancer)	42 ± 1

^*^IC_50_: concentration of a drug required to reduce cell growth by 50%. Cells were treated with 2-AAPA for 6 days. Cell survival rates were determined by the MTT assay. IC_50_ values were derived from corresponding dose–response curves. The data are presented as the mean ± SD of three independent experiments.

### Protein *S*-glutathionylation induced by 2-AAPA

**Figure 2:**
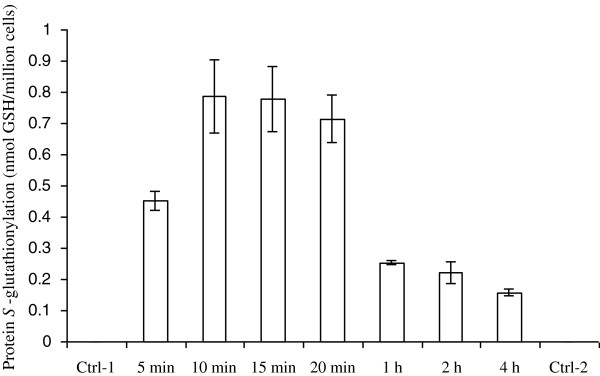
**2-AAPA induces protein *S*-glutationylation in UACC-62 cells.** UACC-62 cells were treated with 50 μM 2-AAPA for different time periods and protein *S*-glutathionylation was determined by the HPLC method described by Chen et al. [16]. The results were presented as the mean ± SD of triplet determination of one set of samples. No protein *S*-glutathionylation was detected in untreated cells. Control 1 and control 2 were conducted at 5 min and 4 h respectively. No *S*-glutathionylation was detected in both control 1 and control 2.

Previously, protein *S*-glutathionylation was detected in both CV-1 (monkey kidney cells) and OVCAR-3 cells treated with 100 μM 2-AAPA [16,17]. Before checking whether microtubules were *S*-glutathionylated in UACC-62 cells, we first determined whether proteins were *S*-glutathionylated in the presence of 2-AAPA. UACC-62 cells were treated with 100 μM 2-AAPA for 20 min and protein *S*-glutathionylation was determined as reported [16]. Protein *S*-glutathionylation (2.19 ± 0.30 nmol GSH/million cells) was detected in UACC-62 cells. The amount of protein *S*-glutathionylation by 2-AAPA induced in UACC-62 cells was similar to that obtained from OVCAR-3 cells confirming 2-AAPA’s ability to induce cellular protein *S*-glutathionylation. UACC-62 cells were then treated with 50 μM 2-AAPA, a concentration close to the IC_50_ value, to determine the time profile of protein *S-*glutathionylation. The results are presented in Figure 2. Protein *S*-glutathionylation was observed at 5 min and reached the maximum in 10 min. At 1 h, protein *S-*glutathionylation was found to be about 40% of the maximum and continued to fall afterwards (Figure 2). 

### Determination of microtubule *S-*glutathionylation

Due to the quantity limitation of microtubules in cells, our HPLC method for protein *S-*glutathionylation was not sensitive enough to detect microtubule *S*-glutathionylation. Microtubule *S*-glutathionylation was thus investigated by a pull-down assay reported by Cheng and co-workers [22] with modification.

**Figure 3:**
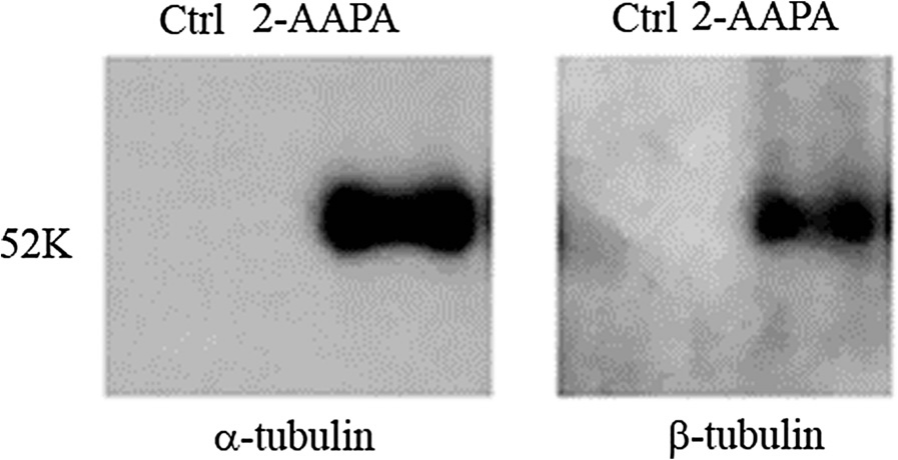
**2-AAPA induces tubulin *S*-glutathionylation.** UACC-62 cells were treated with 100 μM 2-AAPA for 20 min and *S*-glutathionylated proteins were captured by a pull-down assay using GST-agarose beads followed by Western blot analysis. α- and β-Tubulins were detected as described in the method.

Cheng and co-workers used biotinylated glutathione *S*-transferase (GST) from *Schistosoma japonicum* to detect the glutathione moiety of *S-*glutathionylated protein. Since biotinylated GST was not commercially available, we decided to use GST-agarose beads containing the GST from the same species as an alternative. *S*-Glutathionylated proteins were pulled down by GST-agarose beads followed by the Western blot analysis with the antibodies of α-tubulin and β-tubulin. Figure 3 shows that both α-tubulin and β-tubulin were *S*-glutathionylated when UACC-62 cells were treated with 100 μM 2-AAPA for 20 min while no *S*- glutathionylated tubulins were detected in the controls. The densities of the bands are consistent with the fact that α-tubulin has more thiol groups than β-tubulin.

### Effects on microtubule structure

**Figure 4:**
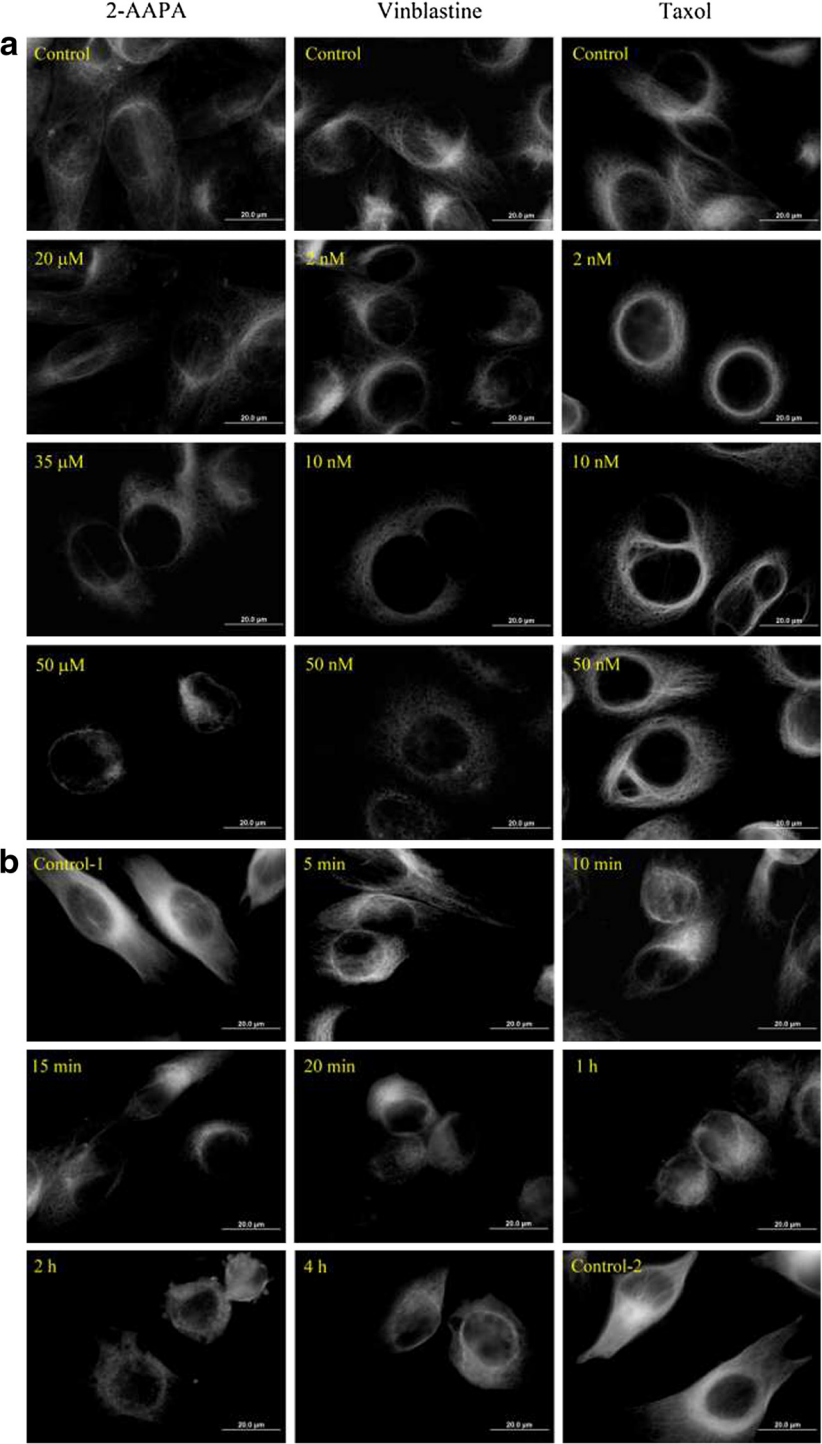
**2-AAPA induces microtubule depolymerization in UACC-62 cells.** UACC-62 cells were treated with the indicated concentrations of 2-AAPA for 12 h (**a**), or with 50 μM 2-AAPA for indicated time periods (**b**), followed by fixation, permeabilization and indirect immunofluorescent analysis with an anti-α-tubulin-FITC. Nuclei were stained with DAPI. Paclitaxel and vinblastine were employed as positive controls for microtubule stabilization and microtubule depolymerization respectively. The data are derived from one of the three independent experiments.

To confirm that microtubule structure was affected by the *S-*glutathionylation, the microtubule network was examined *via* immunofluorescence microscopy. As shown in Figure 4a, microtubules formed an intact network with fine mesh and filaments in untreated cells, while a significant reduction of microtubule density was observed in 2-AAPA-treated cells. Microtubules in treated cells were found dispersed and lost their original linear filamentous structure with disorganized central networks. To compare the effects with that of vinblastine, a microtubule depolymerizing antimitotic drug, and that of paclitaxel, a microtubule stabilizing antimitotic drug, the same experiments were conducted with vinblastine and paclitaxel. Figure 4a shows that the effect of 2-AAPA on microtubules was similar to that of vinblastine revealing that 2-AAPA depolymerized microtubules. The time-dependent effect of 2-AAPA on microtubule structure was further determined by treating UACC-62 cells with 50 μM 2-AAPA for different time periods (Figure 4b). Significant microtubule depolymerization was observed at 5 min. The deploymerization reached a maximum in 20 min. Recovery was observed at one hour. The time profile of microtubule depolymerization (Figure 4b) appears to match well with that of protein *S*-glutathionylation (Figure 2).

### Cell morphological change

**Figure 5:**
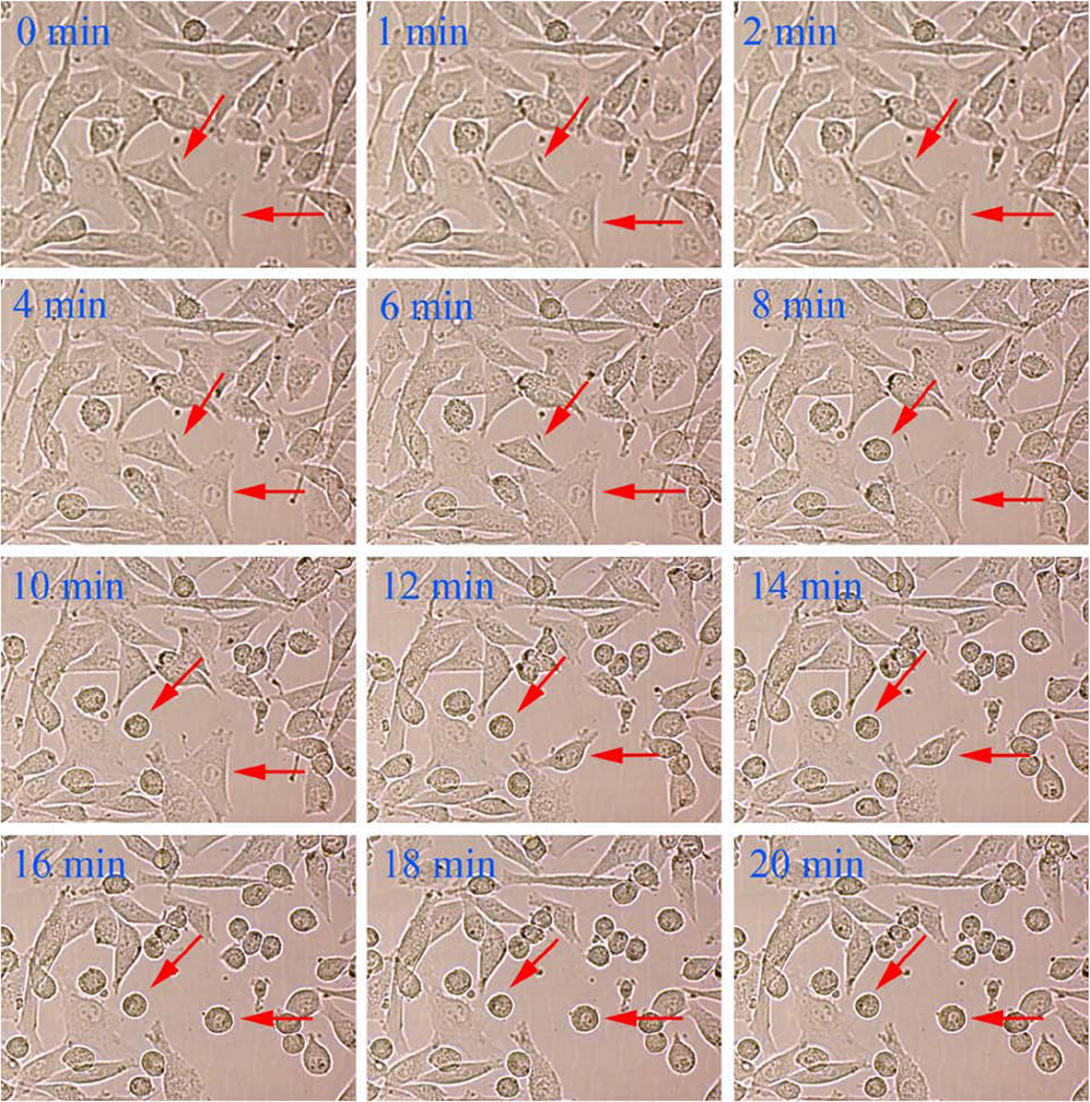
**Effect of 2-AAPA on cell morphological changes in UACC-62 cells.** UACC-62 cells were treated with 50 μM 2-AAPA. Some of the cells undergoing morphological changes are marked by red arrows.

Consistent with the microtubule network being affected, the morphology of the cells was found to be changed. When UACC-62 cells were treated with 50 μM 2-AAPA, the morphology changes were observed as early as 6 min and involved more than 90% cells at the end of 20 min (Figure 5).

### Effects on the cell cycle

**Figure 6:**
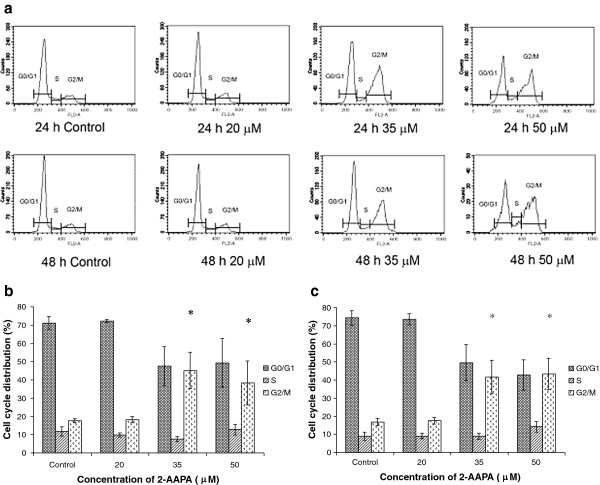
**Effect of 2-AAPA on cell cycle distribution in UACC-62 cells.** The histograms of UACC-62 cells treated with different concentrations of 2-AAPA for 24 h and 48 h are presented (**a**). The bar presentations reflects the quantification of cell cycle distribution at 24 h (**b**) and 48 h (**c**) respectively. Results are presented as the mean ± SD of three independent experiments. *, *P* < 0.05 indicates statistical significance in 2-AAPA treated groups as compared to the control.

To further confirm microtubule function is being affected, the effect on the cell cycle was investigated. After being treated with 2-AAPA (35 and 50 μM), UACC-62 cells were found to be arrested in the G_2_/M phase (Figure 6) which is consistent with microtubule depolymerization.

### Effects on apoptosis

**Figure 7:**
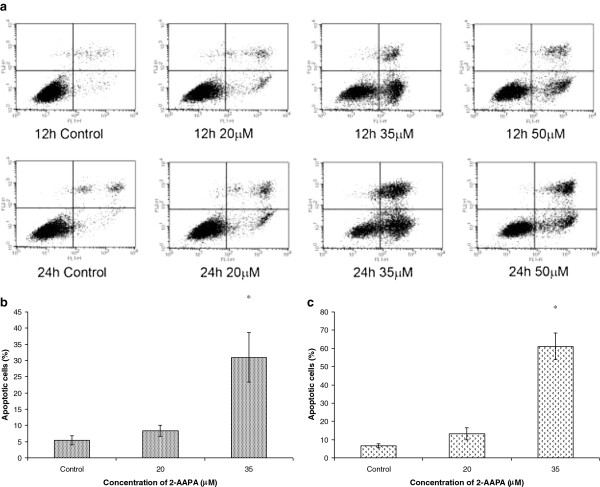
**Effect of 2-AAPA on the induction of apoptosis in UACC-62 cells.** UACC-62 cells were treated with different concentrations of 2-AAPA for 12 h and 24 h and stained with Annexin V and PI. Apoptotic cells were analyzed by flow cytometry (**a**). The percentages of apoptotic cells are presented for 12 h (**b**) and 24 h (**c**) treatment. Results are presented as the mean ± SD of three independent experiments. *, *P* < 0.05 indicates statistical significance in 2-AAPA treated groups as compared to the control.

All anti-microtubule agents identified to date are known to induce apoptotic cell death in cancer cells [23]. We evaluated the effect on apoptosis by treating UACC-62 cells with different concentrations of 2-AAPA for 12 h and 24 h (Figure 7). At 20 μM and 35 μM, 2-AAPA induced apoptosis in a dose- and time-dependent manner (*P* <0.05). 

## Discussion

Antimitotic agents which induce tumor cell mitotic arrest and apoptosis are one of the most important classes of cancer chemotherapeutic agents [24]. However, the clinical application of the current antimitotic drugs is hampered by the problems of undesired side effects, poor pharmacokinetic properties, complex chemical structures, and emerging drug resistance [24]. The emerging drug resistance is of particular concern since it is the major cause of cancer treatment failure. Extensive efforts have been made to search for agents which can effectively interfere with microtubule function, especially those with a novel mechanism of action [25,26].

In this study, we have demonstrated that microtubules can be *S-*glutathionylated. In view of the critical role of the thiol functional groups in microtubule polymerization, it is reasonable to suggest that the microtubule *S-*glutathionylation led to microtubule depolymerization. To confirm this, a correlation study between protein *S*-glutathionylation and microtubule depolymerization was conducted. Ideally, the correlation study should be conducted between microtubule *S*-glutathionylation and microtubule depolymerization. As indicated earlier, the analytical method for the quantification of protein *S*-glutathionylation is not sensitive enough to quantify microtubule *S*-glutathionylation. As presented in the result section, there was a clear correlation between protein *S-*glutathionylation and microtubule depolymerization. The time profile of microtubule depolymerization matched well with that of protein *S*-glutathionylation supporting that microtubule *S-*glutathionylation caused microtubule depolymerization. It is noted that protein *S-*glutathionylation increased initially in the first 10 min and then subsided after 20 min. Similar phenomena were observed earlier in CV-1 cells and believed to be a result of a recovered glutathione reductase activity [17]. The malfunction of microtubules was also reflected in the cell morphology and cell cycle distribution; cells were found to experience morphological changes, arrest at G_2_/M phase and undergo apoptosis.

Protein *S*-glutathionylation has been reported to be a reversible oxidative modification of PSH as a response of the cell to oxidative stress [27-31]. As indicated earlier, protein *S-*glutathionylation involves a mixed disulfide bond (P-S-S-G) formation between PSH and GSH. Once oxidative stress is over, the P-S-S-G will be reduced back to PSH enzymatically by glutaredoxin (Grx) [9,27,29,32-34]. The rich thiol content of microtubules likely makes them susceptible to *S*-glutathionylation. Although the exact mechanism by which 2-AAPA produces protein *S*-glutathionylation, including microtubule *S*-glutathionylation, is not clear, our earlier data showed that protein *S*-glutathionylation induced by 2-AAPA correlates with the formation of the oxidized form of glutathione (GSSG) [35]. Consistently, Carletti and co-workers [36] recently reported that an increase in GSSG in neuron cells produced significant tubulin *S*-glutathionylation. They also determined that tubulin *S*-glutathionylation inhibited tubulin polymerization. Along the same line, we also found that an increase in intracellular GSSG, achieved through delivery of GSSG into cells by a liposome delivery method, produced significant protein *S*-glutathionylation and similar microtubule depolymerization in NCI-H226 cells as observed in this investigation (unpublished data from this laboratory). Taken together, we believe that microtubule *S*-glutathionylation causes microtubule depolymerization. The malfunction of microtubules led to cell morphological changes, cell cycle arrest at G_2_/M phase, and eventually apoptosis.

Finally, the cancer growth inhibition effects of 2-AAPA against other human cancer cell lines were determined. The IC_50_ determination reveals that 2-AAPA inhibited these cell lines with similar IC_50_ values which are close to the concentrations that affect microtubule function suggesting that microtubule *S*-glutathionylation is likely the contribution to cancer cell growth inhibition.

## Conclusions

In summary, our investigation provides evidence demonstrating that microtubule *S*-glutathionylation will lead to microtubule depolymerization, cell cycle arrest at G_2_/M phase, and apoptosis. This mechanism of action is different than those of currently used antimitotic agents. This finding suggests that microtubule *S*-glutathionylation can be potentially used as a novel approach to develop new classes of antimitotic agents which can be complimentary to the existing antimitotic agents.

## Competing interests

The authors declare that they have no competing interests.

## Authors’ contributions

WC carried out the overall studies and drafted the manuscript. TS and YZ helped with the synthetic work and cytotoxicity evaluation of 2-AAPA. AY and XZ helped with the flow cytometric analysis of cell cycle distribution and apoptosis assay. JR provided valuable suggestion on microtubule structure determination. RK provided valuable suggestion on the experiment of immunofluorescence microscopy. XG was responsible for the design and supervision of the study, and preparation of the manuscript. All authors have read and approved the final manuscript.

## Pre-publication history

The pre-publication history for this paper can be accessed here:

http://www.biomedcentral.com/1471-2407/12/245/prepub

## References

[B1] Sharp DJ Rogers GC Scholey JM Microtubule motors in mitosis Nature 2000 407 41 47 10.1038/35024000 10993066

[B2] Kanthou C Tozer GM The tumor vascular targeting agent combretastatin A-4-phosphate induces reorganization of the actin cytoskeleton and early membrane blebbing in human endothelial cells Blood 2002 99 2060 2069 10.1182/blood.V99.6.2060 11877280

[B3] Dustin P Microtubules, 2nd totally rev. edn 1984 Berlin; New York: Springer

[B4] Raff EC The role of multiple tubulin isoforms in cellular microtubule function 1994 New York: John Wiley & Sons

[B5] Zhou J Giannakakou P Targeting microtubules for cancer chemotherapy Curr Med Chem Anticancer Agents 2005 5 65 71 10.2174/1568011053352569 15720262

[B6] Nogales E Wolf SG Downing KH Structure of the alpha beta tubulin dimer by electron crystallography Nature 1998 391 199 203 10.1038/34465 9428769

[B7] Desai A Mitchison TJ Microtubule polymerization dynamics Annu Rev Cell Dev Biol 1997 13 83 117 10.1146/annurev.cellbio.13.1.83 9442869

[B8] Jordan MA Wilson L Microtubules as a target for anticancer drugs Nat Rev Cancer 2004 4 253 265 10.1038/nrc1317 15057285

[B9] Landino LM Moynihan KL Todd JV Kennett KL Modulation of the redox state of tubulin by the glutathione/glutaredoxin reductase system Biochem Biophys Res Commun 2004 314 555 560 10.1016/j.bbrc.2003.12.126 14733943

[B10] Cowan NJ Tubulin genes and the diversity of microtubule function Oxf Surv Eukaryot Genes 1984 1 36 60 6400775

[B11] Britto PJ Knipling L Wolff J The local electrostatic environment determines cysteine reactivity of tubulin J Biol Chem 2002 277 29018 29027 10.1074/jbc.M204263200 12023292

[B12] Britto PJ Knipling L McPhie P Wolff J Thiol-disulphide interchange in tubulin: kinetics and the effect on polymerization Biochem J 2005 389 549 558 10.1042/BJ20042118 15743274 PMC1175133

[B13] Landino LM Brown CM Edson CA Gilbert LJ Grega-Larson N Wirth AJ Lane KC Fluorescein-labeled glutathione to study protein S-glutathionylation Anal Biochem 2010 402 102 104 10.1016/j.ab.2010.02.006 20156418 PMC2883778

[B14] Huber K Patel P Zhang L Evans H Westwell AD Fischer PM Chan S Martin S 2-[(1-methylpropyl)dithio]-1H-imidazole inhibits tubulin polymerization through cysteine oxidation Mol Cancer Ther 2008 7 143 151 10.1158/1535-7163.MCT-07-0486 18202017

[B15] Ducki S Antimitotic chalcones and related compounds as inhibitors of tubulin assembly Anticancer Agents Med Chem 2009 9 336 347 10.2174/1871520610909030336 19275525

[B16] Chen W Zhao Y Seefeldt T Guan X Determination of thiols and disulfides via HPLC quantification of 5-thio-2-nitrobenzoic acid J Pharm Biomed Anal 2008 48 1375 1380 10.1016/j.jpba.2008.08.033 18926658 PMC2684446

[B17] Zhao Y Seefeldt T Chen W Wang X Matthees D Hu Y Guan X Effects of glutathione reductase inhibition on cellular thiol redox state and related systems Arch Biochem Biophys 2009 485 56 62 10.1016/j.abb.2009.03.001 19272349 PMC2709784

[B18] Klatt P Lamas S Regulation of protein function by S-glutathiolation in response to oxidative and nitrosative stress Eur J Biochem 2000 267 4928 4944 10.1046/j.1432-1327.2000.01601.x 10931175

[B19] Townsend DM S-glutathionylation: indicator of cell stress and regulator of the unfolded protein response Mol Interv 2007 7 313 324 10.1124/mi.7.6.7 18199853 PMC6361142

[B20] Seefeldt T Zhao Y Chen W Raza AS Carlson L Herman J Stoebner A Hanson S Foll R Guan X Characterization of a novel dithiocarbamate glutathione reductase inhibitor and its use as a tool to modulate intracellular glutathione J Biol Chem 2009 284 2729 2737 19049979 10.1074/jbc.M802683200 PMC2631970

[B21] Bonifacino JS Dell’Angelica EC Springer TA Immunoprecipitation Curr Protoc Neurosci 2006 Chapter 5 Unit 5 24. 5.24.1-5.24.28 10.1002/0471142301.ns0524s35 18428640

[B22] Cheng G Ikeda Y Iuchi Y Fujii J Detection of S-glutathionylated proteins by glutathione S-transferase overlay Arch Biochem Biophys 2005 435 42 49 10.1016/j.abb.2004.12.016 15680905

[B23] Kaur G Hollingshead M Holbeck S Schauer-Vukasinovic V Camalier RF Domling A Agarwal S Biological evaluation of tubulysin A: a potential anticancer and antiangiogenic natural product Biochem J 2006 396 235 242 10.1042/BJ20051735 16489930 PMC1462728

[B24] Kiselyov A Balakin KV Tkachenko SE Savchuk N Ivachtchenko AV Recent progress in discovery and development of antimitotic agents Anticancer Agents Med Chem 2007 7 189 208 10.2174/187152007780058650 17348827

[B25] Schmidt M Bastians H Mitotic drug targets and the development of novel anti-mitotic anticancer drugs Drug Resist Update 2007 10 162 181 10.1016/j.drup.2007.06.003 17669681

[B26] Nagle A Hur W Gray NS Antimitotic agents of natural origin Curr Drug Targets 2006 7 305 326 10.2174/138945006776054933 16515529

[B27] Dalle-Donne I Milzani A Gagliano N Colombo R Giustarini D Rossi R Molecular mechanisms and potential clinical significance of S-glutathionylation Antioxid Redox Signal 2008 10 445 473 10.1089/ars.2007.1716 18092936

[B28] Dalle-Donne I Rossi R Colombo G Giustarini D Milzani A Protein S-glutathionylation: a regulatory device from bacteria to humans Trends Biochem Sci 2009 34 85 96 10.1016/j.tibs.2008.11.002 19135374

[B29] Dalle-Donne I Rossi R Giustarini D Colombo R Milzani A S-glutathionylation in protein redox regulation Free Radic Biol Med 2007 43 883 898 10.1016/j.freeradbiomed.2007.06.014 17697933

[B30] Gallogly MM Mieyal JJ Mechanisms of reversible protein glutathionylation in redox signaling and oxidative stress Curr Opin Pharmacol 2007 7 381 391 10.1016/j.coph.2007.06.003 17662654

[B31] Spadaro D Yun BW Spoel SH Chu C Wang YQ Loake GJ The redox switch: dynamic regulation of protein function by cysteine modifications Physiol Plant 2010 138 360 371 10.1111/j.1399-3054.2009.01307.x 19912563

[B32] Beer SM Taylor ER Brown SE Dahm CC Costa NJ Runswick MJ Murphy MP Glutaredoxin 2 catalyzes the reversible oxidation and glutathionylation of mitochondrial membrane thiol proteins: implications for mitochondrial redox regulation and antioxidant DEFENSE J Biol Chem 2004 279 47939 47951 10.1074/jbc.M408011200 15347644

[B33] Holmgren A Johansson C Berndt C Lonn ME Hudemann C Lillig CH Thiol redox control via thioredoxin and glutaredoxin systems Biochem Soc Trans 2005 33 1375 1377 10.1042/BST20051375 16246122

[B34] Reynaert NL van der Vliet A Guala AS McGovern T Hristova M Pantano C Heintz NH Heim J Ho YS Matthews DE Dynamic redox control of NF-kappaB through glutaredoxin-regulated S-glutathionylation of inhibitory kappaB kinase beta Proc Natl Acad Sci U S A 2006 103 13086 13091 10.1073/pnas.0603290103 16916935 PMC1559757

[B35] Zhao Y Seefeldt T Chen W Carlson L Stoebner A Hanson S Foll R Matthees DP Palakurthi S Guan X Increase in thiol oxidative stress via glutathione reductase inhibition as a novel approach to enhance cancer sensitivity to X-ray irradiation Free Radic Biol Med 2009 47 176 183 10.1016/j.freeradbiomed.2009.04.022 19397999 PMC2745482

[B36] Carletti B Passarelli C Sparaco M Tozzi G Pastore A Bertini E Piemonte F Effect of protein glutathionylation on neuronal cytoskeleton: a potential link to neurodegeneration Neuroscience 2011 192 285 294 21704675 10.1016/j.neuroscience.2011.05.060

